# Plasma Inflammatory Factors Are Associated with Anxiety, Depression, and Cognitive Problems in Adults with and without Methamphetamine Dependence: An Exploratory Protein Array Study

**DOI:** 10.3389/fpsyt.2015.00178

**Published:** 2015-12-18

**Authors:** Marilyn Huckans, Bret E. Fuller, Alison L. N. Chalker, Madeleine Adams, Jennifer M. Loftis

**Affiliations:** ^1^Research and Development Service, VA Portland Health Care System, Portland, OR, USA; ^2^Mental Health and Clinical Neurosciences Division, VA Portland Health Care System, Portland, OR, USA; ^3^Department of Psychiatry, Oregon Health & Science University, Portland, OR, USA; ^4^Methamphetamine Abuse Research Center, Oregon Health & Science University, Portland, OR, USA

**Keywords:** anxiety, biological markers, depression, cognition, cytokines, inflammation, substance abuse

## Abstract

**Objectives:**

It is hypothesized that immune factors influence addictive behaviors and contribute to relapse. The primary study objectives were to (1) compare neuropsychiatric symptoms across adults with active methamphetamine (MA) dependence, in early remission from MA dependence, and with no history of substance dependence, (2) determine whether active or recent MA dependence affects the expression of immune factors, and (3) evaluate the association between immune factor levels and neuropsychiatric symptoms.

**Methods:**

A cross-sectional study was conducted using between group comparisons and regression analyses to investigate associations among variables. Eighty-four adults were recruited into control (CTL) (*n* = 31), MA-active (*n* = 17), or MA-remission (*n* = 36) groups. Participants completed self-report measures of anxiety, depression, and memory complaints and objective tests of attention and executive function. Blood samples were collected, and a panel of immune factors was measured using multiplex technology.

**Results:**

Relative to CTLs, MA-dependent adults evidenced greater anxiety and depression during active use (*p* < 0.001) and remission (*p* < 0.007), and more attention, memory, and executive problems during remission (*p* < 0.01) but not active dependence. Regression analyses identified 10 immune factors (putatively associated with cytokine–cytokine receptor interactions) associated with anxiety, depression, and memory problems.

**Conclusion:**

While psychiatric symptoms are present during active MA dependence and remission, at least some cognitive difficulties emerge only during remission. Altered expression of a network of immune factors contributes to neuropsychiatric symptom severity.

## Introduction

Methamphetamine (MA) addiction is a pressing health concern and is a substance use disorder that faces many treatment challenges, including, but not limited to, the lack of effective treatments and the high prevalence of co-occurring mental and physical health conditions associated with the addiction. According to the 2012 National Survey on Drug Use and Health, approximately 1.2 million people reported using MA in the past year, and 440,000 reported using it in the past month (1). The costs associated with MA use are numerous, with contributions from crime/criminal justice, child endangerment, lost productivity, drug treatment, health care, MA production hazards, and premature death estimated at more than $23.4 billion in 2005 (2).

Exposure to MA impacts a range of both peripheral and central immune functions, such as alterations in B- and T-cell expression and response (e.g., antibody production, proliferation), natural killer cell activation, macrophage function (e.g., phagocytosis), glial cell-mediated cytokine expression, and immune cell trafficking – which likely contribute to the chronicity of the drug’s adverse and neurotoxic effects (3–23). Furthermore, MA use is associated with structural and functional changes to regions of the brain that regulate cognitive and psychiatric function and promote drug-seeking behaviors, making recovery from MA addiction very difficult (24–28). Approximately 40% of chronic MA users experience global neuropsychiatric difficulties (e.g., memory, attention, and executive functions) (26, 29, 30), and one-third to one-half or more of MA-dependent adults evidence psychiatric disorders (e.g., anxiety and depression) during remission (31–35). Neuropsychiatric impairments that persist following abstinence are associated with poorer treatment outcomes, including increased relapse rates, lower treatment retention rates, and reduced daily functioning (36–38).

Research is beginning to link immune factor signaling with neural and behavioral aspects of addiction, such as impaired cognitive function (10), drug seeking behaviors, and resilience to relapse (39–42). The immune system modulates central nervous system functions through a variety of signaling mechanisms, both in the absence and in the presence of immunological challenges (43–46). Likewise, numerous studies have demonstrated that peripheral alterations in the expression and function of immune factors (e.g., pro- and anti-inflammatory cytokines and chemokines) are evident in patients diagnosed with a range of neuropsychiatric and mental health disorders, including depression (47, 48), anxiety (49, 50), chronic fatigue syndrome (51), cancer-related fatigue and cognitive impairment (52), pain disorders (53–56), hepatitis C virus (HCV)-associated neuropsychiatric impairment (57), age-related cognitive decline and dementia (58–60), and, more recently, substance use disorders (10, 61). Collectively, these studies highlight the impact that immune activation and immune factor dysregulation (both peripherally and centrally) can have on central nervous system function.

In a translational study, we previously reported that mice exposed with repeated doses of MA evidence significantly altered expression of brain and plasma immune factors, both immediately following drug exposure and after a remission period, and that altered expression of several plasma immune factors is significantly correlated with reduced cognitive function in humans during remission from MA dependence (10). However, this study was limited to a panel of nine immune factors, and it did not include active MA users who may demonstrate different neuropsychiatric and immune consequences than those in remission from MA dependence. Although research investigating pathophysiological mechanisms and treatment approaches for MA addiction has been expanding, few studies have examined the relationship between immune factor expression and the development of commonly occurring neuropsychiatric symptoms in adults with MA use disorders. The primary objectives of the present study, therefore, were to (1) compare levels of anxiety, depression, and cognitive difficulties across three study groups – adults with active MA dependence, adults in early remission from MA dependence, and adult controls (CTLs) with no history of drug or alcohol dependence, (2) determine whether active or recent MA dependence and other covariates [age, gender, ethnicity, body mass index (BMI), nicotine use, or medical comorbidities] significantly influence the expression of a larger array of immune factors, and (3) evaluate the association between peripheral immune factor expression and anxiety, depression, and cognition in adults with and without MA dependence. The functional significance of the immune factors found to be predictive of neuropsychiatric impairments in our regression models was assessed using bioinformatics pathway analysis.

## Materials and Methods

### Research Participants

Eighty-four research participants gave informed consent, met all eligibility criteria, and were enrolled in the study; 29 others gave informed consent but were not enrolled in the study because they did not meet all eligibility criteria (i.e., they were screen fails). Participants were recruited from Portland area addiction treatment centers and the community through word of mouth and via study advertisements posted in clinics, websites, and newspapers. Participants were recruited into one of three groups: (1) CTL group (*n* = 31): adults with no lifetime history of dependence on any substance other than nicotine or caffeine; (2) MA-active (ACT) group (*n* = 17): adults actively using MA and currently meeting criteria for MA dependence; and (3) MA-remission (REM) group (*n* = 36): adults in remission from MA dependence ≥1 and ≤12 months.

General exclusion criteria included history of a major medical illness or current use of medications that are likely to be associated with serious neurological or immune dysfunction [e.g., stroke, traumatic brain injury, human immunodeficiency virus (HIV) infection, HCV infection, primary psychotic disorder, immunosuppressants, antivirals, anti-tumor necrosis factor (TNF)-alpha agents]. Additional exclusion criteria for the non-dependent CTL group included: (1) meets criteria for lifetime history of dependence on any substance (other than nicotine or caffeine dependence) based on diagnostic and statistical manual of mental disorders-fourth edition (DSM-IV) (62) and confirmed by the Mini-International Neuropsychiatric Interview (MINI) (63) and (2) on the day of the study visits, tests positive on a urine drug analysis for any drug of abuse. Additional inclusion criteria for the MA-ACT group included: (1) meets DSM-IV (62) criteria for dependence on MA, confirmed by the MINI (63), (2) average MA use was ≥2 days/week for ≥1 year (average years of dependence = 13.91, SD = 10.00), and (3) last use of MA was ≤2 weeks ago (average days since last use = 2.25 days, SD = 2.21 days, range = 1–8). Note that all individuals in the MA-ACT group completed a urine drug analysis, and most (58.8%) tested positive for MA. MA is generally detectable by urine drug analysis for approximately 3–5 days depending on a variety of individual factors such as the pH level of the urine. Thus, we included 7 participants (41.2% of the group) who tested negative for MA, reported having last used MA ≥ 3 days ago, and who otherwise met all criteria for the group and study. Although this is a limitation in that we could not verify MA use for those individuals, many individuals use MA only 2–3 days/week, so this procedure allowed us to include a sample that was generalizable to a broader range of use patterns. Additional inclusion criteria for the MA-REM group included: (1) meets DSM-IV (62) criteria for dependence on MA, confirmed by the MINI (63), (2) average MA use ≥2 days/week for ≥1 year (average years of dependence = 11.24, SD = 5.77), (3) last use of MA was ≥1 and ≤12 months ago (average days since last use = 108.82, SD = 73.82, range = 35–273), and (4) on the day of the study visit, does not test positive on a urine drug analysis for MA or any other drugs of abuse.

### Ethical Approval

The protocol conformed to the ethical guidelines of the 1975 Declaration of Helsinki (6th revision, 2008) and was approved by the Institutional Review Boards at the Veterans Affairs Portland Health Care System and Oregon Health & Science University.

### Procedures

Research participants were compensated with grocery store vouchers ($50) to complete the following study procedures: clinical interview, urine drug analysis, HCV and HIV antibody screening, blood sample collection for multiplex immune factor analysis as described in the sections below, questionnaires to assess severity of anxiety, depression and memory complaints, and objective cognitive measures to assess attention and executive function. Given the scope of the study, in order to facilitate rapid enrollment across the 14-month enrollment period, study visits were kept below 90 min; our questionnaire and cognitive assessment battery was limited to 15 min and, thus, did not include a comprehensive battery of objective cognitive tests. All procedures, including blood sample collection, were completed by certified phlebotomist/retired Licensed Practical Nurse (LPN) who was trained and supervised by a licensed psychologist and clinical neuropsychologist (Marilyn Huckans). To ensure accuracy, all measures were scored and then re-scored by separate study personnel, and all data were entered into a database initially and then double-checked by separate study personnel prior to analysis.

#### Questionnaires and Cognitive Assessment Measures

1. General anxiety disorder-7 scale (GAD-7) (64), a well-validated 7-item measure of anxiety.
2. Patient health questionaire-9 (PHQ-9) (65), a well-validated 9-item measure of depression severity.
3. Prospective and retrospective memory questionnaire (PRMQ) (66), a well-validated 16-item measure of self-reported memory complaints.
4. Digits forward, neuropsychological assessment battery (NAB) (67), well-validated and widely used objective performance measure of simple auditory attention span and tracking.
5. Letter fluency, Delis–Kaplan executive functioning system (D-KEFS) (68), a well-validated objective performance measure of verbal fluency and executive function.

### Multiplex Immune Factor Assessments

Following all other study procedures, blood was drawn in the afternoon (mean time was 1:28 p.m., SD = 2:35 h) by one-time venipuncture into cell preparation tubes (BD Vacutainer Systems, Franklin Lakes, NJ, USA) containing 1 mL of 0.1M sodium citrate solution. The blood was then centrifuged at 1500 rpm for 20 min at room temperature (22–25°C). Plasma was separated, collected, and immediately aliquoted in polypropylene tubes (Phenix Research Products, Hayward, CA, USA) and frozen at −80°C until assayed. Samples were sent frozen in a single batch to Myriad Rules Based Medicine, Inc. (Austin, TX, USA) where they were thawed for assay without additional freeze–thaw cycles. Immune factors were measured using Myriad Rules Based Medicine, Inc.’s Human InflammationMAP v 1.0 panel, a biomarker panel designed to discern inflammatory patterns in biological samples including plasma. Myriad Rules Based Medicine, Inc. is a Clinical Laboratory Improvement Amendments (CLIA) certified laboratory. Assays conducted by this company utilizing this methodology have been published previously [e.g., Ref. (57, 69–71)]. This multiplex microbead assay is based on Luminex technology (72) and measures proteins in a similar manner to standard sandwich enzyme-linked immunosorbent assays (ELISAs), with comparable sensitivity and range. Multiplex assays have been compared to regular high sensitivity ELISAs in studies of Alzheimer’s disease, Parkinson’s disease, parasite infection, HIV, and others (73–76). Twenty-one factors from the original panel of 47 factors were undetectable (not measurable on the standard curve) in more than one-third of the total sample and were excluded from analyses [i.e., granulocyte-macrophage colony-stimulating factor, interferon-gamma, interleukin (IL)-1alpha, IL-1beta, IL-1 receptor antagonist, IL-2, IL-3, IL-4, IL-5, IL-6, IL-7, IL-10, IL-12p40, IL-12p70, IL-15, IL-17, macrophage inflammatory protein-1 alpha, matrix metalloproteinase-9, tumor necrosis factor (TNF)-alpha, and TNF-beta]. Table 2 summarizes the 26 remaining immune factors used for our analyses, including the factor’s abbreviation, unit of measurement, and the percentage within the total sample with detectable levels.

### Bioinformatics Pathway Analysis

Functional analysis of the immune factor expression data was performed using the DAVID (Database for Annotation, Visualization and Integrated Discovery v6.7) Bioinformatics Resources,^1^ as previously described, with minor modifications (71). Briefly, the 10 immune factors identified as significant predictors of neuropsychiatric symptoms (Table 3) were assessed for significant enrichment of biological processes using the terms of the fifth level of gene ontology (GO). The name of each factor was converted to an analyzable identifier via Universal Protein Resource (UniProt) and then entered in the DAVID functional annotation tool. Pathway mapping analysis was carried out using the Kyoto Encyclopedia of Genes and Genomes (KEGG) module within DAVID.

### Statistical Analysis

Data analyses were conducted using Stata v.12 (StataCorp LP, College Station, TX, USA), with specific methodologies described in the footnotes for Tables 1–3. Significant *p* values were ≤0.050.

**Table 1:** **Between group comparisons of demographics, clinical characteristics, anxiety, depression, and cognition in adults with and without methamphetamine dependence**.

	CTL	MA-ACT	MA-REM	Ominbus model	Significant comparisons
**Demographics**					
Age, mean years (SD)^a^	37.55 (13.84)	41.24 (10.29)	36.78 (9.46)	*p* = 0.393	n.s.
Male gender^b^	68%	81%	78%	*p* = 0.515	n.s.
Caucasian^b^	81%	76%	81%	*p* = 0.933	n.s.
Years of education, mean (SD)^a^	14.13 (1.41)	12.76 (0.56)	12.08 (1.56)	*F* (9, 74) = 3.45; *p* = 0.001**	MA-ACT < CTL *p* = 0.006** MA-REM < CTL *p* < 0.001***
**Clinical characteristics**					
Body mass index, mean (SD)^a^	29.00 (5.83)	28.27 (6.01)	28.92 (5.54)	*p* = 0.951	n.s.
Current tobacco use^b^	58%	94%	89%	LR *X*^2^ = 12.40; *p* = 0.002**	MA-ACT > CTL *p* = 0.011* MA-REM > CTL *p* = 0.007**
Current prescription medication use^c^	32%	18%	51%	–	MA-REM > MA-ACT *z* = 2.39 *p* < 0.02*
Past medical diagnoses (any)^b^	52%	47%	58%	*p* = 0.717	n.s.
Past psychiatric diagnosis (any)^b^^,^^d^	45%	41%	61%	*p* = 0.277	n.s.
**Neuropsychiatric symptom severity**					
Anxiety (GAD-7), mean total score (SD)^e^	2.42 (3.21)	8.42 (6.62)	5.44 (5.21)	LR *X*^2^ = 12.16; *p* < 0.002***	MA-ACT > CTL *z* = 3.49 *p* < 0.001*** MA-REM > CTL *z* = 2.72 *p* < 0.001***
Depression (PHQ-9), mean total score (SD)^e^	2.61 (3.14)	8.41 (6.39)	5.47 (5.43)	LR *X*^2^ = 12.56; *p* < 0.002**	MA-ACT > CTL *z* = 3.56 *p* < 0.001*** MA-REM > CTL *z* = 2.69 *p* < 0.007**
Memory complaints (PRMQ), mean total score (SD)^e^	28.84 (7.59)	34.47 (6.55)	37.11 (11.71)	*F* = 6.51; *p* = 0.002**	MA-REM > CTL *z* = 3.58 *p* < 0.001***
Attention (NAB digits forward), mean *z* score (SD)^a^	0.31 (1.05)	−0.007 (0.98)	−0.33 (1.05)	*F* = 3.37; *p* = 0.039*	MA-REM < CTL *z* = −2.59 *p* < 0.001***
Executive function/verbal fluency (D-KEFS), mean *z* score (SD)^a^	0.46 (0.85)	0.10 (1.06)	−0.92 (0.74)	*F* = 3.54; *p* = 0.034*	MA-REM < CTLS *z* = −2.64 *p* = 0.010**

*^a^For continuous variables with normal distributions, results reflect linear regressions with two dummy codes to reflect the membership across the three groups and test the group differences between the two drug conditions compared to controls. The *p* values reflect each dummy code testing two null hypotheses: MA-ACT = CTLs and MA-REM = CTLs*.

*^b^For binary variables, *p* values reflect the results of logistic regressions with two dummy codes to test two null hypotheses: MA-ACT = CTLs and MA-REM = CTLs*.

*^c^Medication use was compared between groups with tests of proportions*.

*^d^Psychiatric diagnoses were based on diagnostic and statistical manual of mental disorders-fourth edition (DSM-IV) criteria verified using the Mini-International Neuropsychiatric Interview (MINI)*.

*^e^For non-normal distributions, *p* values reflect results from negative binomial regressions with two dummy codes for group membership. The *p* values reflect each dummy code testing two null hypotheses: MA-ACT = CTLs and MA-REM = CTLs*.

*CTL, adults with no history of drug or alcohol dependence based on DSM-IV criteria verified with the MINI; D-KEFS, Delis–Kaplan executive function system; GAD-7, generalized anxiety disorder 7-item scale; MA-ACT, adults meeting DSM-IV criteria for active methamphetamine dependence, verified with the MINI; MA-REM, adults meeting DSM-IV criteria for remission from methamphetamine dependence, verified with the MINI; NAB, neuropsychological assessment battery; n.s., non-significant (*p* > 0.050); PHQ-9, patient health questionnaire; PRMQ, prospective and retrospective memory questionnaire; PTSD, posttraumatic stress disorder*.

*p ≤ 0.050.

**p ≤ 0.010.

***p ≤ 0.001.

**Table 2:** **Covariate analysis of plasma immune factor levels in adults with and without methamphetamine dependence**.

Immune factor (abbreviation)	Units	Total sample % with detectable factors^a^ (%)	CTLs mean; median; SD^b^	MA-ACT mean; median; SD^b^	MA-REM mean; median; SD^b^	Omnibus model^c^	Significant variable^d^
Alpha-2-macroglobulin (A2Macro)	mg/mL	100	0.98; 1.00; 0.27	0.91; 0.86; 0.41	0.97; 0.95; 0.21	*F* (8, 74) = 4.46; *p* = 0.001***	MA-ACT (*t* = −2.39; *p* = 0.019)*
							Age (*t* = −2.63; *p* = 0.010)*
							Male (*t* = −3.19; *p* = 0.002)*
Alpha-1-antitrypsin (AAT)	mg/mL	100	0.95; 0.94; 0.23	1.05; 1.10; 0.18	1.01; 0.97; 0.38	*F* (8, 74) = 0.80; *p* > 0.050	
Beta-2-microglobulin (B2M)	μg/mL	100	0.82; 0.80; 0.24	1.07; 1.00; 0.35	1.00; 0.87; 0.47	*F* (8, 74) = 2.34; *p* = 0.027*	MA-REM (*t* = 2.00; *p* = 0.050)*
							Caucasian (*t* = −2.21; *p* = 0.030)*
Brain-derived neurotrophic factor (BDNF)	ng/mL	100	1.91; 1.70; 0.80	2.36; 2.20; 1.17	2.52; 2.20; 1.30	*F* (8, 74) = 1.39; *p* > 0.050	
C-reactive protein (CRP)	μg/mL	100	2.87; 1.30; 3.42	1.46; 0.91; 1.70	2.13; 0.75; 3.18	*F* (8, 74) = 3.74; *p* = 0.001**	BMI (*t* = 4.75; *p* < 0.000)***
Complement C3 (C3)	mg/mL	100	0.65; 0.62; 0.16	0.64; 0.60; 0.13	0.70; 0.62; 0.41	*F* (8, 74) = 0.71; *p* > 0.050	
Eotaxin-1	pg/mL	95	210.81; 200.00; 73.38	242.42; 207.00; 96.92	274.08; 243.30; 116.13	*F* (8, 74) = 3.83; *p* = 0.001***	MA-REM (*t* = 2.30; *p* = 0.024)*
							Caucasian (*t* = 2.44; *p* = 0.017)*
							BMI (*t* = −3.10; *p* = 0.003)**
Factor VII	ng/mL	100	331.23; 323.00; 84.27	358.41; 370.00; 80.32	310.11; 289.50; 99.03	*F* (8, 74) = 3.39; *p* = 0.002**	Age (*t* = 2.70; *p* = 0.009)**
							Male (*t* = −3.65; *p* < 0.000)***
Ferritin	ng/mL	100	61.17; 41.00; 57.60	88.96; 64.00; 72.28	68.50; 53.50; 63.80	*F* (8, 74) = 3.71; *p* = 0.001*	Male (*t* = 3.70; *p* < 0.001)***
							Caucasian (*t* = −2.55; *p* = 0.013)*
Fibrinogen	mg/mL	100	2.34; 2.20; 0.61	2.25; 2.20; 0.50	2.41; 2.25; 0.92	*F* (8, 74) = 1.82; *p* > 0.050	
Haptoglobin	mg/mL	95	0.85; 0.77; 0.36	1.16; 1.00; 0.50	0.90; 0.81; 0.48	*F* (8, 74) = 1.72; *p* > 0.050	
Intercellular adhesion molecule 1 (ICAM-1)	ng/mL	100	79.71; 75.00; 23.48	78.24; 77.00; 25.09	79.53; 78.50; 18.58	*F* (8, 74) = 1.19; *p* > 0.050	
Interleukin-8 (IL-8)	pg/mL	77	4.67; 4.30; 3.55	3.98; 4.50; 2.41	3.85; 3.90; 3.20	*F* (8, 74) = 3.74; *p* = 0.001**	BMI (*t* = 2.11; *p* = 0.038)**
							Age (*t* = 3.80; *p* < 0.000)***
Interleukin-18 (IL-18)	pg/mL	100	135.68; 133.00; 64.48	118.94; 111.00; 56.54	131.92; 140.00; 44.51	*F* (8, 74) = 1.33; *p* > 0.050	
Interleukin-23 (IL-23)	ng/mL	79	0.40; 0.46; 0.24	0.53; 0.53; 0.16	0.35; 0.42; 0.30	*F* (8, 74) = 2.35; *p* = 0.026*	Male (*t* = −2.47; *p* = 0.016)*
							Caucasian (*t* = −2.06; *p* = 0.043)*
							Age (*t* = 2.07; *p* = 0.042)*
Macrophage inflammatory protein-1 beta (MIP-1 beta)	pg/mL	99	83.58; 79.00; 37.60	70.36; 64.00; 30.38	78.94; 70.50; 66.94	*F* (8, 74) = 1.19; *p* > 0.050	
Matrix metalloproteinase-3 (MMP-3)	ng/mL	100	6.17; 4.90; 2.73	9.39; 8.40; 4.57	7.83; 6.35; 5.66	*F* (8, 74) = 2.65; *p* = 0.013*	Male (*t* = 3.19; *p* = 0.002)**
Monocyte chemotactic protein 1 (MCP-1)	pg/mL	100	182.48; 172.00; 55.42	190.47; 200.00; 57.38	203.08; 186.50; 81.64	*F* (8, 74) = 0.47; *p* > 0.050	
Regulated on activation, normal T cell expressed and secreted (RANTES)	ng/mL	100	6.78; 6.80; 2.82	8.32; 7.50; 3.73	8.15; 7.25; 3.71	*F* (8, 74) = 1.66; *p* > 0.050	
Stem cell factor (SCF)	pg/mL	99	158.52; 157.00; 45.56	178.29; 182.00; 39.93	169.00; 163.50; 78.58	*F* (8, 74) = 1.28; *p* > 0.050	
Tissue inhibitor of metalloproteinases 1 (TIMP-1)	ng/mL	100	38.58; 37.00; 7.96	42.65; 43.00; 5.51	41.11; 41.00; 7.97	*F* (8, 74) = 1.37; *p* > 0.050	
Tumor necrosis factor receptor 2 (TNFR2)	ng/mL	100	2.49; 2.40; 0.85	2.71; 2.80; 0.86	2.84; 2.40; 1.30	*F* (8, 74) = 0.87; *p* > 0.050	
Vascular cell adhesion molecule-1 (VCAM-1)	ng/mL	100	260.52; 261.00; 46.56	258.06; 263.00; 62.10	265.56; 259.50; 69.47	*F* (8, 74) = 0.28; *p* > 0.050	
Vascular endothelial growth factor (VEGF)	pg/mL	99	59.94; 62.00; 17.81	67.18; 63.00; 18.17	71.58; 70.00; 28.91	*F* (8, 74) = 0.98; *p* > 0.050	
Vitamin D-binding protein (VDBP)	μg/mL	100	159.16; 160.00; 62.07	140.24; 138.00; 55.21	158.36; 152.50; 100.52	*F* (8, 74) = 1.50; *p* > 0.050	
Vascular endothelial growth factor (VEGF)	pg/mL	99	59.94; 62.00; 17.81	67.18; 63.00; 18.17	71.58; 70.00; 28.91	*F* (8, 74) = 0.98; *p* > 0.050	
von Willebrand factor (vWF)	μg/mL	100	38.03; 36.00; 16.81	44.15; 41.00; 19.15	39.97; 36.50; 15.52	*F* (8, 74) = 2.45; *p* = 0.020*	BMI (*t* = 2.88, *p* = 0.005)**

*^a^Percentage of adults within the total sample with detectable immune factor levels*.

*^b^The means, medians, and SD of immune factor levels within each group are reported*.

*^c^*p* Values reflect results of linear regressions with group (two dummy to test the following null hypotheses: MA-ACT = CTL and MA-REM = CTL) and covariates [age, race, male gender, Caucasian race, body mass index (BMI), nicotine use, and any current medical condition] regressed onto each immune factor level*.

*^d^Group membership and covariates were evaluated when the omnibus model was significant (*p* < 0.050), and they were reported as significant variables in the table when *p* < 0.050 for the variable*.

*p ≤ 0.050.

**p ≤ 0.010.

***p ≤ 0.001.

**Table 3:** **Multi-analyte regression models^a^**.

	Anxiety			Depression			Memory complaints		

Model fit	LR *X*^2^ = 68.29; *p* = 0.0004			LR *X*^2^ = 80.29; *p* < 0.0001			*F* (34, 48) = 2.41; *p* = 0.0026		

Variable	*b*	*z*	*p*	*b*	*z*	*p*	*b*	*t*	*p*
Intercept	1.07	0.63	0.527	0.073	0.06	0.954	32.97	2.27	0.027*
MA-ACT	1.50	3.28	0.001**	1.04	3.24	0.001***	5.75	1.52	0.135
MA-REM	−0.52	1.43	0.153	0.45	1.67	0.095	9.85	3.23	0.002**
Age	−0.022	−1.58	0.115	−0.0049	−0.54	0.588	−1.37	−1.23	0.223
Caucasian	−0.059	−0.13	0.894	−0.073	−0.24	0.813	5.27	1.51	0.138
Male	−0.27	−0.62	0.536	−0.702	−2.25	0.024*	−0.045	−0.01	0.991
BMI	−0.37	−1.41	0.158	0.033	1.69	0.091	0.012	0.05	0.958
Nicotine	0.45	1.14	0.254	0.34	1.16	0.247	2.74	0.82	0.419
Past medical diagnosis	−0.079	−0.32	0.752	0.16	0.86	0.388	0.64	0.30	0.767
AAT	0.46	0.59	0.553	−0.12	−0.22	0.828	−12.31	−1.84	0.072
A2Macro	0.22	0.33	0.744	0.60	1.22	0.224	−2.51	−0.44	0.660
B2M	−0.46	−0.84	0.401	−0.44	−1.12	0.265	−3.10	−0.67	0.507
BDNF	−0.0062	0.04	0.965	−0.09	0.92	0.356	−1.99	−1.47	0.147
CRP	0.16	2.46	0.014*	0.027	0.63	0.529	−1.00	−2.08	0.043*
C3	−1.39	−0.86	0.392	−0.0097	−0.01	0.993	−11.04	−1.04	0.302
Eotaxin-1	−0.0039	−2.28	0.022*	−0.003	−2.42	0.015*	−0.039	−3.14	0.003**
Factor VII	−0.00084	0.46	0.648	−0.015	−1.13	0.260	0.011	0.68	0.499
Ferritin	−0.0041	−1.65	0.099	−0.00015	0.09	0.932	−0.18	−0.87	0.390
Fibrinogen	−0.57	−0.13	0.899	−0.69	−2.18	0.029*	−1.50	−0.41	0.681
Haptoglobin	0.32	0.85	0.397	1.04	3.93	<0.001***	3.77	1.17	0.249
ICAM-1	−0.014	−1.96	0.051	−0.015	−2.80	0.005**	−0.022	−0.35	0.729
IL-8	0.14	−2.45	0.014*	0.12	2.94	0.003**	0.14	0.32	0.747
IL-18	0.0044	1.83	0.067	0.0018	1.00	0.316	0.021	0.94	0.352
IL-23	−2.03	−3.27	0.001***	−1.023	2.28	0.023*	−1.28	−0.24	0.812
MIP-1 beta	0.00094	0.34	0.732	0.0016	0.89	0.373	0.007	0.33	0.745
MMP3	0.11	2.92	0.004**	0.071	2.66	0.008**	0.49	1.51	0.139
MCP1	−0.002	−1.27	0.204	−0.0017	−1.13	0.259	0.015	0.98	0.333
SCF	0.013	4.03	<0.001***	0.0096	4.26	<0.001***	0.014	0.53	0.598
RANTES	0.045	0.89	0.376	0.045	1.27	0.204	0.31	0.67	0.506
TIMP1	−0.026	−0.95	0.345	0.0088	0.41	0.683	0.27	1.10	0.277
TNFR2	−0.31	−1.36	0.173	−0.077	−0.46	0.648	0.23	0.12	0.906
VCAM1	0.0032	1.02	0.307	0.0008	0.34	0.734	−0.025	−1.00	0.324
VEGF	0.015	2.36	0.018*	0.0058	1.34	0.182	0.045	0.78	0.439
VDBP	0.001	0.40	0.690	0.00084	0.51	0.613	0.017	0.85	0.402
VWF	−0.019	−1.50	0.134	−0.013	−1.41	0.159	0.052	0.52	0.604

*^a^Regression models were developed in order to determine whether the panel of plasma immune markers was significantly predictive of each of the five neuropsychiatric outcome variables (i.e., anxiety, depression, memory complaints, attention, and executive function) within the total sample. For each neuropsychiatric outcome variable, regressions were estimated by a Maximum Likelihood function with assumptions of normal or negative binomial distributions depending on the shape of the outcome variable. Normal distributions were assumed for the attention, memory and executive function outcomes, and negative binomial distributions were assumed for the anxiety and depression variables. Models were calculated (shown in the table above) by simultaneously regressing the set of covariates and immune factors on each outcome variable. For each of the final regression models, fit parameters are presented as well as the unstandardized regression weights (*b*), *z*, or *t* values (depending on type of regression used) and *p* values for each immune marker. Positive *b* weights for the immune marker meant that scores on the neuropsychiatric outcome variable increased with the increase in the immune marker; those with negative values decreased when the neuropsychiatric variable increased. Results are not shown for the attention or executive function models because the overall models were not significant (*p* > 0.050). See Table 2 for immune factor abbreviations*.

*p ≤ 0.050.

**p ≤ 0.010.

***p ≤ 0.001.

## Results

### Demographics and Clinical Data

Table 1 summarizes demographic data, clinical characteristics, MA use characteristics, and neuropsychiatric outcomes by study group. Groups differed significantly by education (on average CTLs had two more years of education than the MA groups), tobacco use (a lower proportion of CTLs used tobacco), and current medications (the MA-ACT group was significantly less likely to be taking any medications than the MA-REM group, but there were no significant differences between MA-ACT and CLTs or MA-REM and CTLs). There were no significant group differences in terms of age, gender, race, BMI, or rates of medical or psychiatric diagnoses; adults with severe or unstable medical or psychiatric disorders were excluded from the study. MA-ACT and MA-REM groups reported significantly higher levels of anxiety and depression than CTLs. Relative to CTLs, the MA-REM group, but not the MA-ACT group, reported more memory problems and performed worse on tests of attention and executive function.

### Covariate Analyses of Plasma Immune Markers

Table 2 summarizes the results of regression analyses to determine if study group or covariates (age, race, gender, BMI, tobacco use, and any medical condition) significantly predicted each of the peripheral immune factor levels in the total sample. Each of the regression models had a single Type I error rate for the predictors (*p* < 0.05) that was determined by the omnibus test of the model fit, limiting the risk of Type I error due to multiple comparisons. Being in the MA-ACT group versus CTL group was significantly associated with lower levels of A2Macro, and being in the MA-REM group versus the CTL group was significantly associated with higher levels of B2M and eotaxin-1. Older age was significantly associated with lower levels of A2Macro and higher levels of factor VII, IL-23, and IL-8. Male gender was significantly associated with lower levels of A2Macro, factor VII, and IL-23, and higher levels of ferritin and MMP-3. Caucasian race was significantly associated with lower levels of B2M, ferritin, and IL-23, and higher levels of eotaxin-1. BMI was significantly associated with higher levels of CRP, IL-8, and vWF, and lower levels of eotaxin-1. Neither using tobacco nor having a medical condition was significantly associated with any immune factor level.

### Immune Factor Predictors of Anxiety, Depression, and Cognition

**Figure 1:**
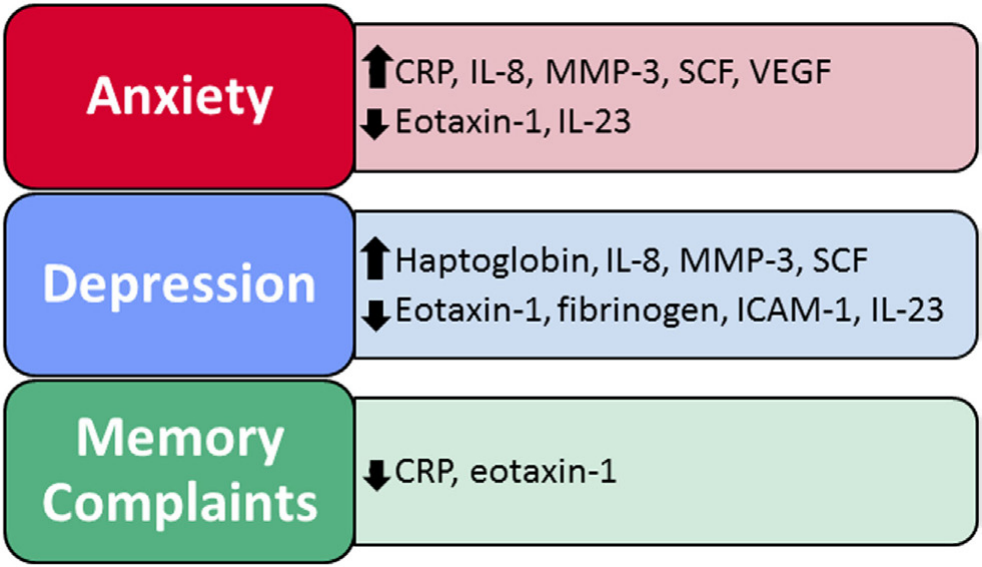
**Immune factors predict self-reported anxiety, depression, and memory problems**. Two to eight immune factors were significant predictors of neuropsychiatric function within each of the regression models, for a total of 10 significant immune factors across all models.

As summarized in Table 3, across all participants, regressions (controlling for study group and age, race, gender, BMI, tobacco use, and any medical condition as covariates) revealed a direct relationship between plasma immune factor levels and anxiety, depression, and memory complaints; the overall regression models were not significant for attention or executive function and so were not included in the table. Each of the regression models had a single Type I error rate for the predictors that was determined by the omnibus test of the model fit, limiting the risk of Type I error due to multiple comparisons. Figure 1 summarizes results from the regression models and shows the immune factors that were found to be significantly associated with each neuropsychiatric outcome. A total of 10 variables were found to be significantly associated with neuropsychiatric outcomes (i.e., CRP, eotaxin-1, fibrinogen, haptoglobin, ICAM-1, IL-8, IL-23, MMP-3, SCF, and VEGF).

### Bioinformatic Functional Analysis

Using the Kyoto Encyclopedia of Genes and Genomes (KEGG) pathways analysis feature of the database for annotation, visualization, and integrated discovery (DAVID), we analyzed the 10 immune factors found to be significantly associated with neuropsychiatric symptoms in Table 3 for significant interaction within biologically relevant pathways. In doing so, the cytokine–cytokine receptor interaction pathway was determined to be most relevant (*p* = 0.0002), incorporating five (i.e., eotaxin-1, IL-8, IL-23, SCF, and VEGF) of the previously identified 10 immune factors (Figure S1 in Supplementary Material; Table 4). Four other pathways – bladder cancer (*p* = 0.056), pathways in cancer (*p* = 0.07), NOD-like receptor signaling pathway (*p* = 0.082), and complement and coagulation (*p* = 0.091) – were also generated by the database; however, they did not reach statistical significance.

**Table 4:** **Immune and neuromodulatory effects of inflammatory factors associated with neuropsychiatric symptomatology**.

Factor^a^	Immune and neuromodulatory effects	Reference
CRP	Protein produced by the liver and released into blood plasma in acute response to injury (and therefore inflammation). It serves to activate the complement system, promoting phagocytosis. Higher levels of CRP have been associated with a greater risk of dementia, microstructural white matter damage, and poorer scores on executive function tests, but there are also findings that lower levels of CRP and other mediators of inflammation are associated with moderate alcohol use	(90–93)
*Eotaxin-1	Small CC chemokine (a.k.a. CCL-11) involved in eosinophil chemotaxis. Studies in mice have related early exposure to eotaxin-1 to decreased cognitive performance and hippocampal neurogenesis. In humans, serum levels of eotaxin-1 are increased in schizophrenic and late-stage bipolar patients as compared to controls	(94–96)
Fibrinogen	Large glycoprotein involved in the formation of blood clots. While normally blocked by the blood brain barrier (BBB), infiltration of fibrinogen into the brain, such as in the case of Alzheimer’s disease, has been linked with an exacerbation of vascular damage. Reduced levels of fibrinogen are associated with moderate alcohol consumption, as has been reported for other inflammatory biomarkers (e.g., CRP, ICAM-1)	(91, 92, 97, 98)
Haptoglobin	Acute-phase reactant protein that binds free hemoglobin when it is released from erythrocytes. Elevated plasma haptoglobin levels are observed in an animal model of neuropsychiatric lupus (mice display increased depressive-like behaviors and memory deficits)	(99)
ICAM-1	Transmembrane protein present in leukocytes and endothelial cells, the concentration of which increases upon cytokine stimulation – for example, following inflammatory pain. In the case of acute inflammation-related pathology (i.e., ischemia, autoimmune disorders, and encephalitis) the rise in ICAM-1 strongly correlates with increased BBB permeability. Exposure to substances of abuse can result in differential effects on circulating levels of ICAM-1. For example, moderate alcohol consumption is associated with significantly lower serum ICAM-1 levels, as compared to both heavy alcohol consumption and no consumption (abstinence)	(100–103)
*IL-8	Macrophage-produced chemokine that attracts neutrophils, basophils, and T-cells. The use of MA has been shown to increase LPS-mediated IL-8 expression in human macrophages. In one study, CSF IL-8 expression post TBI was 1000 times higher than plasma IL-8 levels, suggesting that it plays a prominent role in modulating neuroinflammation	(104–107)
*IL-23	Heterodimeric cytokine is composed of an IL-23p40 subunit and an IL-23p19 subunit. A decline in serum IL-23 levels is observed in animal models of stress-induced depression, and the reduction in IL-23 is associated with a reduced proportion of T helper 17 cells with decreased proliferation capacity	(108)
MMP-3	Stromelysin endopetidase involved in extracellular matrix remodeling. High concentrations of MMP-3 are seen in the case of severe TBI, where the elevated MMP-3 is associated with inflammation, BBB disruption, bleeding, and cell death	(109–111)
*SCF	Cytokine that plays a role in hematopoiesis, spermatogenesis, and melanogenes. SCF is overexpressed by neurons and glial following brain injury and has been shown to produce an angiogenic response *in vivo* in part by recruiting progenitor cells to damaged sites	(112, 113)
*VEGF	Signaling protein and a subfamily of growth factors involved in vasculogenesis as well as angiogenesis. VEGF overexpression drives BBB leakage in the case of ischemia, and elevated serum levels of VEGF are observed in animal models of stress-related brain disorders	(114–116)

^a^See Table 2 for a list of immune factor abbreviations. Factors with an asterisk (*) were identified by the KEGG pathway analyses as having functional relevance within the cytokine–cytokine receptor interaction pathway (Figure S1 in Supplementary Material).

## Discussion

This cross-sectional analysis of adults with and without a history of MA dependence indicates that, compared with non-dependent CTLs, both active MA users and those in recent remission from MA dependence report significantly (*p* < 0.050) higher levels of anxiety and depression (Table 1). Notably, while adults in remission from MA dependence reported significantly more memory problems and performed worse on attention and executive function tasks than CTLs, active MA users did not evidence these cognitive deficits relative to CTLs (Table 1). While numerous studies have documented cognitive impairments in adults in remission from MA dependence (10, 26, 29, 30), relatively few have examined cognitive function in non-intoxicated adults during active dependence (i.e., non-remission). Our findings suggest that pathological processes that occur following abstinence from MA dependence, rather than during active use *per se*, result in at least some cognitive problems that initially appear and persist during remission.

Results additionally suggest that current and recent MA dependence, age, gender, race, and BMI can significantly impact peripheral immune signaling, as they were each significant predictors of some but not all plasma immune factor levels in our sample (Table 2). This is consistent with the previous literature showing altered peripheral immune signaling in substance users (10, 61), older adults (59), women (77), black Americans (78), and adults with higher BMI (79).

Moreover, results show that differences in the expression of a network of peripheral immune proteins significantly impact neuropsychiatric function in adults both with and without present or past MA dependence. In our regression models (which controlled for covariates that might impact neuropsychiatric function or immune factor expression – MA dependence, age, gender, race, BMI, nicotine use, and medical comorbidities), a subset of immune factors significantly predicted self-reported anxiety, depression, and memory problems (Table 3). Specifically, two to eight immune factors were significant predictors of neuropsychiatric function within each of the regression models, for a total of 10 significant immune factors across all models – CRP, eotaxin-1, fibrinogen, haptoglobin, ICAM-1, IL-8, IL-23, MMP-3, SCF, and VEGF (Figure 1).

A major goal of this study was to identify novel biomarkers that might be relevant to the treatment of neuropsychiatric symptoms, including the context of MA use disorder, and these 10 significant predictors are thus potentially worthy of further investigation through additional studies (e.g., as treatment targets). It is notable that each of these factors have both immunoregulatory and neuromodulatory functions (see Table 4 for a summary of their actions), with the potential to both enhance inflammatory responses and adversely impact neuronal functions (e.g., induce microstructural white matter damage, impair phagocytosis, alter cell trafficking, and compromise BBB integrity) when dysregulated. Thus, our results suggest that efforts to develop and investigate novel immunotherapies as treatments for neuropsychiatric symptoms (particularly in the context of substance use disorders) are warranted.

In animal models of MA addiction and other substance use disorders, a variety of addictive behaviors (e.g., withdrawal, craving, conditioned place preference, locomotor sensitization, self-administration, sedation, and motor impairments) have been significantly altered through interventions that directly impact immune signaling (80, 81). Consequently, immunotherapies that are designed to simultaneously “normalize” immunoregulation and neuromodulation may be particularly effective in treating neuropsychiatric symptoms that can contribute to and exacerbate substance abuse. Clinical trials have already demonstrated the antidepressant benefits of several immunotherapies, such as etanercept (TNF-alpha antagonist used to treat a range of autoimmune conditions, including psoriasis and arthritis), infliximab (monoclonal antibody against TNF-alpha also used for the treatment of autoimmune diseases), and celecoxib [cyclooxygenase (COX)-2 inhibitor used to treat pain and arthritis] (82, 83). Additional immunotherapies for the treatment of neuropsychiatric and substance use disorders are currently under investigation by our lab and others. For example, our recent preclinical efficacy data demonstrate that RTL551, a partial major histocompatibility construct (pMHC) and novel neuroimmune modulator, effectively reduces MA-induced memory deficits in mice (84) and regulates the expression of pro-inflammatory cytokines (84), indicating that the immune system modulates the pharmacodynamics and behavioral consequences of drug actions. Similarly, ibudilast (AV-411 or MN-166), a less specific peripheral and central anti-inflammatory agent, reduces MA self-administration and stress-induced relapse in rodents (85–87) and is currently being evaluated as a potential neuroimmune therapy for adults with MA, opioid, or alcohol use disorders.^2^

The present study includes several limitations. A cross-sectional study design does not allow for definitive conclusions on causality, and group sample sizes may have limited our statistical power. Regression analyses are considered exploratory in nature and should be interpreted cautiously prior to replication. Our sample was largely middle aged, male, and Caucasian, with all adults residing in one Northwest metropolitan area (the greater Portland area), so results may not be generalizable to more diverse populations. Our study was limited to a panel of peripheral plasma immune proteins; additional biomarkers and functional pathways of interest may be revealed by future human studies, including gene arrays, or through animal studies that are better able to evaluate brain tissue for central immune factor expression. Our study included only a brief battery of questionnaires and cognitive assessment measures. Although we did not find a significant relationship between our immune factors and our two objective cognitive measures (i.e., digit span and fluency), we did find a significant relationship between our immune factors and self-reported memory complaints. Future studies could, therefore, include a more comprehensive battery of objective cognitive tests to determine whether immune factors are more predictive of subjective cognitive complaints versus objective cognitive performance. Lastly, it should be noted that this study was exploratory in nature (i.e., included a large array of immune factors without *a priori* hypotheses about each) and should be replicated before results are deemed definitive.

Despite limitations, our results demonstrate that, relative to non-dependent CTL participants, MA-dependent adults evidence anxiety and depressive symptoms both during active use and remission, and they experience difficulties with aspects of cognition (attention, memory, and executive function) that initially develop and then persist only during remission – an observation with noteworthy treatment implications (88). To the best of our knowledge, this is one of first studies to investigate the role of inflammatory immune factors on neuropsychiatric symptoms in adults with active MA dependence, as compared to both adults in remission from MA and to non-dependent CTLs. Results suggest that altered expression of a network of plasma immune factors contributes to neuropsychiatric symptom severity (i.e., anxiety, depression, and memory problems) in adults with and without MA addiction. Moreover, our study identified 10 immune factors (CRP, eotaxin-1, fibrinogen, haptoglobin, ICAM-1, IL-8, IL-23, MMP-3, SCF, and VEGF) that may be particularly relevant to neuropsychiatric symptoms, given their putative roles in cytokine–cytokine receptor interactions (Figure S1 in Supplementary Material) and in the regulation of both immune and neuronal functions (Table 4). Thus, although the pathophysiological mechanisms contributing to MA addiction are not fully understood, immune dysregulation and immune factors, such as cytokines, chemokines, and cellular adhesion molecules, likely play a critical role in perpetuating MA-induced neuronal injury and neuropsychiatric impairments (10, 89).

## Author Contributions

The work presented here was carried out in collaboration among all authors. JL and MH designed the research plan and methods. MH supervised the research participant procedures. JL directed the laboratory work. BF, MH, and JL analyzed the data, interpreted the results and wrote the paper. AC and JL conducted the DAVID analyses. MA and AC performed literature searches, contributed to writing the paper, and discussed analyses, interpretation, and presentation. All authors have contributed to, seen and approved the manuscript.

## Conflict of Interest Statement

The authors have the following conflicts: Oregon Health & Science University, the VA Portland Health Care System, Dr. Marilyn Huckans and Dr. Jennifer M. Loftis have a significant financial interest in Artielle Immunotherapeutics, Inc., a company that may have a commercial interest in the results of this research and technology. These potential individual and institutional conflicts of interest have been reviewed and managed by Oregon Health & Science University.

## Footnotes

^1^https://david.ncifcrf.gov/home.jsp

^2^www.clinicaltrials.gov
