# Supplementary material for: Plasma Inflammatory Factors Are Associated with Anxiety, Depression, and Cognitive Problems in Adults with and without Methamphetamine Dependence: An Exploratory Protein Array Study
Source: Front Psychiatry. 2015 Dec 18;6:178. doi: 10.3389/fpsyt.2015.00178 (PMC4683192; doi:10.3389/fpsyt.2015.00178)
Supplement: Supplementary file 1 [file Data_Sheet_1.DOCX]

**Supplementary Material**

**Legend for Supplementary Figure 1**

The cytokine-cytokine receptor interaction signaling pathways as represented in the Kyoto Encyclopedia of Genes and Genomes (KEGG; <http://www.genome.jp/kegg/kegg1.html>) (1-5). We identified a subset of 10 immune factors that significantly predicted self-reported anxiety, depression, and memory problems. Using KEGG pathway analyses, these 10 factors were evaluated for significant interactions within biologically relevant pathways, and the cytokine-cytokine receptor interaction pathway was determined to be most relevant (p = 0.0002), incorporating five [*i.e*., eotaxin-1 (CCL-11), IL-8, IL-23, SCF (KIT ligand), and VEGF] of the previously identified 10 immune factors. Connections represent relations between adjacent molecules, with red stars identifying the five significant immune factors (see also **Table 4**).

**Supplementary References**

1. Huang da W, Sherman BT, Lempicki RA. Systematic and integrative analysis of large gene lists using DAVID bioinformatics resources. Nat Protoc. 2009;4:44–57.
2. Huang da W, Sherman BT, Lempicki RA. Bioinformatics enrichment tools: paths towards the comprehensive functional analysis of large gene lists. Nucleic Acids Res. 2009;37:1–13.
3. Kanehisa M, Goto S. KEGG: Kyoto encyclopedia of genes and genomes. Nucleic Acids Res. 2000;28:27–30.
4. Kanehisa M, Goto S, Hattori M, Aoki-Kinoshita KF, Itoh M, Kawashima S, et al. From ge-nomics to chemical genomics: new developments in KEGG. Nucleic Acids Res. 2006;34:D354–D357.
5. Kanehisa M, Goto S, Furumichi M, Tanabe M, Hirakawa M. KEGG for representation and analysis of molecular networks involving diseases and drugs. Nucleic Acids Res. 2010;38:D355–D360.
