# Supplementary figures and images for: Plasma Inflammatory Factors Are Associated with Anxiety, Depression, and Cognitive Problems in Adults with and without Methamphetamine Dependence: An Exploratory Protein Array Study
Source: Front Psychiatry. 2015 Dec 18;6:178. doi: 10.3389/fpsyt.2015.00178 (PMC4683192; doi:10.3389/fpsyt.2015.00178)

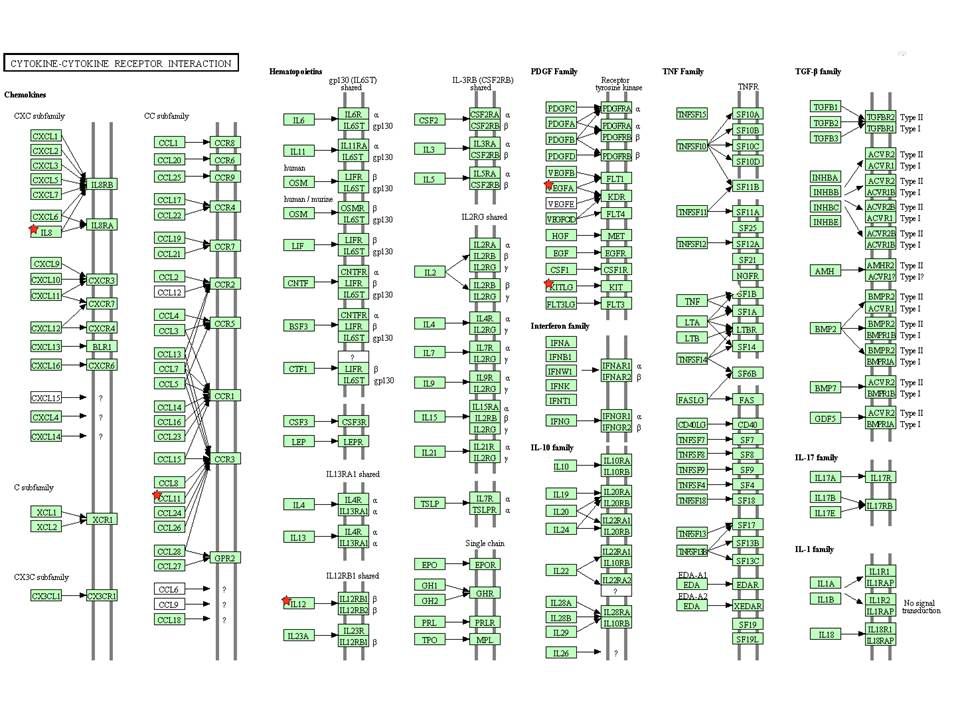

Supplement: Supplementary file 2 [file Image_1.JPEG]
